# A detailed analysis of F-MuLV- and SFFV-infected cells in Friend virus-infected mice reveals the contribution of both F-MuLV- and SFFV-infected cells to the interleukin-10 host response

**DOI:** 10.1186/s12977-022-00613-4

**Published:** 2022-12-16

**Authors:** Philip Podschwadt, Anna Malyshkina, Sonja Windmann, Tanja Werner, Wiebke Hansen, Wibke Bayer

**Affiliations:** 1 Institute for Virology, University Hospital Essen, University Duisburg-Essen, Essen, Germany; 2 Institute for Medical Microbiology, University Hospital Essen, University Duisburg-Essen, Essen, Germany

**Keywords:** Friend virus, Friend murine leukemia virus, Spleen focus forming virus, Interleukin-10, Erythroleukemia

## Abstract

**Background:**

Friend virus (FV) is a complex of the Friend murine leukemia virus (F-MuLV) and the replication-defective, pathogenic spleen focus forming virus (SFFV). In the past, we used a fluorescently labeled F-MuLV to analyze FV target cells. To build on these findings, we have now created a double-labeled FV that contains a Katushka-labeled F-MuLV and an mTagBFP-labeled SFFV, which we have used to study the infection by the two individual viruses in the FV infection of highly susceptible BALB/c mice.

**Results:**

Our data show that the target cells of SFFV largely mirror those of F-MuLV, with the highest virus loads in erythroblasts, B cells and myeloid cells. The early phase of infection was dominated by cells infected by either SFFV or F-MuLV, whereas double-infected cells became dominant later in the course of infection with increasing viral loads. In the late phase of infection, the frequency of double-infected cells was similarly high as the frequencies of SFFV or F-MuLV single-infected cells, and single- and double-infected cells outnumbered the uninfected cells in the most highly infected cell populations such as erythroblasts. FV and retroviruses in general have been shown to induce interleukin 10 (IL-10) as a means of suppressing immune responses. Interestingly, we found in infected IL-10-eGFP reporter mice that SFFV-infected cells contributed to the IL-10-producing cell pool much more significantly than F-MuLV-infected cells, suggesting that the truncated SFFV envelope protein gp55 might play a role in IL-10 induction. Even though BALB/c mice mount notoriously weak immune responses against FV, infection of mice with an ablation of IL-10 expression in T cells showed transiently lower viral loads and stronger T cell activation, suggesting that IL-10 induction by FV and by SFFV in particular may contribute to a suppressed immune response in BALB/c mice.

**Conclusion:**

Our data provide detailed information about both F-MuLV- and SFFV-infected cells during the course of FV infection in highly susceptible mice and imply that the pathogenic SFFV contributes to immune suppression.

**Supplementary Information:**

The online version contains supplementary material available at 10.1186/s12977-022-00613-4.

## Background

Friend virus (FV) is a pathogenic murine retrovirus complex that was first described by Charlotte Friend in 1957 as an infectious agent with the ability to induce erythroleukemia in adult mice [1]. It has since been recognized to be a complex of Friend murine leukemia virus (F-MuLV) and spleen focus forming virus (SFFV; [2]), with the latter being responsible for the pathogenicity of the FV complex. The pathogenicity depends on a range of murine host factors (reviewed in [3]), the most important for erythroleukemia development being the factor Friend virus susceptibility 2 (*Fv-2*). The susceptible allele of *Fv-2* (*Fv-2*^*s*^) encodes a truncated form of the stem cell-derived tyrosine kinase receptor (Stk), referred to as sf-Stk [4]. Interaction of the SFFV Env protein gp55 with the erythropoietin receptor and with sf-Stk results in the activation of signal transduction pathways that lead to proliferation of erythroid cells (reviewed in [5]). Two different strains of SFFV have been described that differ in their pathogenic effect: anemia-inducing SFFV (SFFV_A_) leads to anemia due to hemodilution, in contrast to polycythemia-inducing SFFV (SFFV_P_) (reviewed in [6]). In both cases, the infected mice develop typical splenomegaly and erythroblastosis, with proliferating proerythroblasts being rendered erythropoietin-independent (SFFV_P_) or hypersensitive to erythropoietin (SFFV_A_) [7–10].

The existing literature provides little information on the frequency of single- and double-infected cells in FV-infected mice. In fact, many publications focus exclusively on either one of the two viruses of the FV complex, depending on the aim of the research. F-MuLV is regarded as the immunologically more relevant component of the complex, since it provides the viral proteins that are used by both viruses due to large deletions in all open reading frames of SFFV [11], and contains CD8^+^ and CD4^+^ T cell epitopes that are recognized by T cells in infected mice, which can be readily detected [12–14]. Vaccines against FV based on peptides or proteins [15–17], as well as gene-based vaccines such as DNA-based vectors [18–20], vaccinia virus-based vectors [21, 22], mouse cytomegalovirus- [23] and adenovirus-based vectors [20, 24–28] always rely on the induction of immune responses against F-MuLV. SFFV, on the other hand, is the pathogenic component of the FV complex, and studies of erythroleukemia development have focused on this virus and its effect on cells of the erythroblast lineage [29–33].

In a previous study, we used an FV complex containing F-MuLV labeled with the fluorescent protein mWasabi and characterized the infected target cell population both in FV-resistant and susceptible mice [34]. In this previous work, we showed that erythroblasts, myeloid cells, B cells and T cells carried the bulk of the viral load in the acute phase of infection, while follicular B cells, follicular CD4^+^ T cells and macrophages carried the highest viral loads and served as a virus reservoir in the chronic phase of infection of genetically resistant mice. Follicular B and T cells are assumed to be protected from CD8^+^ T cell killing due to the location in the B cell follicle that CD8^+^ T cells usually cannot enter because of a lack of the appropriate chemokine receptors, as shown in HIV and SIV infection (reviewed in [35]). Macrophages have been shown to be less susceptible to cytotoxic T cell killing in HIV infection [36], and inhibitory molecules such as serpin serine protease inhibitor 6 have been implied in this resistance [37].

While work with FV-mWasabi provided important insights into the target cells of FV, the infection with this virus only allowed the analysis of F-MuLV-infected but not SFFV-infected cells. Therefore, we created a new fluorescent virus complex in which both F-MuLV and SFFV are labeled with the fluorescent proteins Katushka [38] and mTagBFP [39], respectively. This new virus complex allows for the easy detection of F-MuLV or SFFV single-infected and F-MuLV and SFFV double-infected cells. Since SFFV plays an important role in the pathogenicity of the FV complex, we performed infection experiments in mice that are genetically susceptible to FV induced erythroleukemia and studied the distribution of both viruses in different target cells over time. Switching to the red fluorescent protein Katushka for the labeling of F-MuLV furthermore allowed the use of this virus complex for the infection of reporter mice that express eGFP. Because IL-10 has been suggested in the past to play a role in the immune response to FV infection [27, 40], we were interested in the induction of this immunosuppressive cytokine and performed infection experiments in IL-10-eGFP reporter mice.

## Results

### Construction of a bright fluorescently double-labeled FV complex

**Fig. 1:**
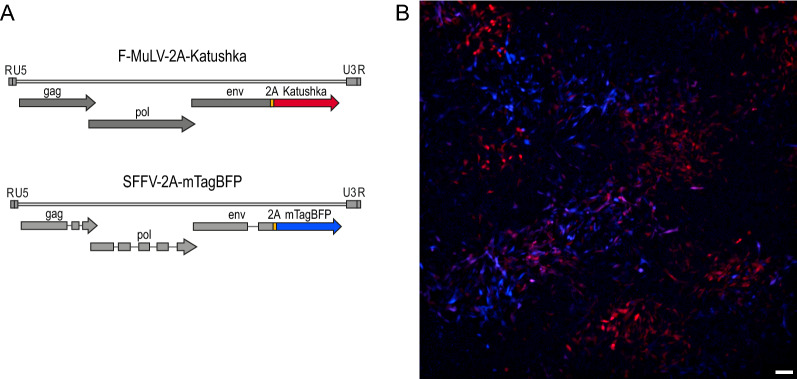
Design of the fluorescently labeled viruses. **A** The fluorescently labeled complex FV-Katushka-mTagBFP consists of Katushka-encoding F-MuLV (top) and mTagBFP-encoding SFFV (bottom). The open reading frames for the fluorescent proteins were fused to the envelope open reading frames via a 2A self-cleaving peptide. **B** Fluorescence microscopy image of *M. dunni* fibroblast cells infected with FV-Katushka-mTagBFP; red: Katushka, blue: mTagBFP; white bar: 100 μm

To expand on our previous findings on the target cells of FV, where we used a fluorescently labeled FV complex in which an mWasabi-labeled F-MuLV was complexed with wild-type SFFV, we now constructed a double-labeled FV complex to allow the individual analysis of both F-MuLV- and SFFV-infected cells in infected mice. To facilitate a reliable distinction of the two viruses by flow cytometry, we selected the red fluorescent protein Katushka (excitation maximum λ_ex_: 588 nm, emission maximum λ_em_: 635 nm) for the labeling of F-MuLV and the blue fluorescent protein mTagBFP (λ_ex_: 402 nm, λ_em_: 457 nm) for the labeling of SFFV. Using non-green fluorescent proteins would furthermore allow for the use of widely used eGFP reporter mice. Both viruses were obtained by the fusion of the fluorescent protein open reading frames to the envelope open reading frames via a self-cleaving 2 A peptide (Fig. 1A), which we showed previously to lend stability to the modified virus [34]. The resulting viruses were successfully reconstituted into a complex, and fluorescence was readily detected by both flow cytometry (data not shown) and fluorescence microscopy (Fig. 1B).

### F-MuLV- and SFFV-infected target cell frequencies in BALB/c mice

**Fig. 2:**
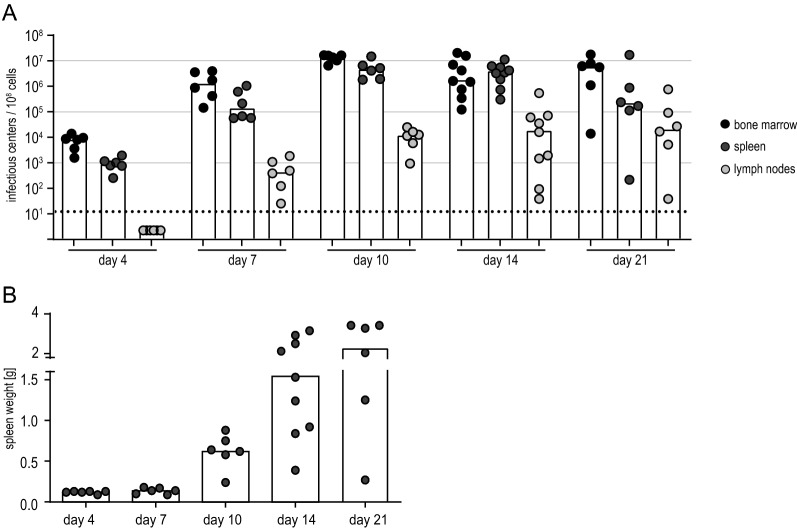
Infectious center frequencies in FV-Katushka-mTagBFP in BALB/c mice. Highly susceptible BALB/c mice were infected with 500 SFFU of FV-Katushka-mTagBFP, and viral loads in the bone marrow, lymph nodes and spleen on days 4, 7, 10, 14 and 21 (**A**) and the spleen weights (**B**) were analyzed. Each dot indicates an individual mouse, bars indicate the median (**A**) or mean values (**B**). The dotted line indicates the detection limit. Data from 6 (days 4, 7, 10, 21) or 9 (day 14) mice per group were obtained in 1–3 independent experiments per time point

To characterize the FV-Katushka-mTagBFP complex and to analyze infected cells, we infected BALB/c mice and isolated bone marrow, spleens and lymph nodes at different time points after infection. When we analyzed the viral loads by conventional infectious center assay [41], we found that viral loads in the bone marrow and spleen, and to lesser degree in lymph nodes, rose rapidly and reached high levels by day 10 after infection (Fig. 2A), and the mice developed the typical FV-induced splenomegaly [42] (Fig. 2B). Compared to infection with unmodified FV, infection with FV-Katushka-mTagBFP resulted in comparable levels of infectious centers in the bone marrow, spleen and lymph nodes with a trend toward lower levels in FV-Katushka-mTagBFP infected mice (Additional file 1: Fig. S1A). The development of splenomegaly as observed by spleen weight was delayed in mice infected with FV-Katushka-mTagBFP compared to mice infected with unmodified FV, with significantly lower spleen weights on days 10 and 14 (Additional file 1: Fig. S1B). The frequency of erythroblasts in spleen cells, which increases during FV infection of susceptible mice due to the proliferation induced by SFFV gp55, was significantly lower in FV-Katushka-mTagBFP infected mice compared to mice infected with unmodified FV on day 10 but reached comparable levels by day 14 (Additional file 1: Fig. S1C). These results indicate that the replicative capacity of FV-Katushka-mTagBFP may be slightly reduced compared to the unmodified FV, but the overall pathogenicity of the complex, while delayed, was preserved, as shown by the expansion of the splenic erythroblast population and development of severe splenomegaly.

**Fig. 3:**
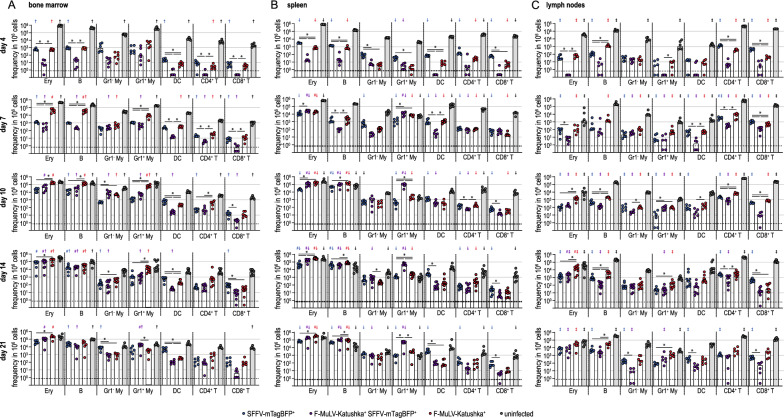
F-MuLV- and SFFV-infected cells in infected BALB/c mice. Highly susceptible BALB/c mice were infected with 500 SFFU of FV-Katushka-mTagBFP, and infected erythroblasts (Ery), B cells, Gr1^−^ (Gr1^−^ My) and Gr1^+^ myeloid cells (Gr1^+^ My), dendritic cells (DC), CD4^+^ T cells and CD8^+^ T cells in the bone marrow (**A**), spleen (**B**) and lymph nodes (**C**) were analyzed by flow cytometry on days 4, 7, 10, 14 and 21. The frequencies of F-MuLV-Katushka single-infected, SFFV-mTagBFP single-infected and F-MuLV-Katushka, SFFV-mTagBFP double-infected as well as uninfected cells are shown as indicated. Each dot indicates an individual mouse, bars indicate median values, the dotted line indicates the detection limit. Data from 6 (days 4, 7, 10, 21) or 9 (day 14) mice per group were obtained in 1–2 independent experiments per time point. Asterisk indicates statistically significant differences between the indicated types of single or double-infected cells (*p* < 0.05, multiple t-test with Bonferroni correction for multiple comparisons). Number sign indicates dominant subsets of single or double-infected cells in the respective organ, i.e. infected cell types that were significantly higher than at least three other cell types of the same single or double-infected state (*p* < 0.05, Kruskal–Wallis ANOVA on Ranks, Dunn’s post test). Dagger indicates statistically significant differences between the respective cell subsets in the bone marrow and spleen, double dagger indicates statistically significant differences between the respective cell subsets in the bone marrow and lymph nodes, inverted cross symbol indicates statistically significant differences between the respective cell subsets in the spleen and lymph nodes (*p* < 0.05, multiple t-test with Bonferroni correction for multiple comparisons)

We next analyzed the target cells expressing the F-MuLV-Katushka and SFFV-mTagBFP proviruses in FV-Katushka-mTagBFP-infected mice by high-parameter flow cytometry (Fig. 3). While we only detected those infected cells that actively express an integrated provirus, for simplicity, we will refer to these provirus-expressing cells as infected cells when describing our results. At the early time point of day 4 after FV-Katushka-mTagBFP infection, the highest frequencies of infected cells were detected in erythroblasts and B cells in the spleen (Fig. 3B), whereas in the bone marrow, Gr1^−^ and Gr1^+^ myeloid cells were also infected at frequencies comparable to those of infected erythroblasts and B cells (Fig. 3A). In lymph nodes, we already found more substantial frequencies of infected CD4^+^ and CD8^+^ T cells at these early time points (Fig. 3C), which seemingly reflects these cell types’ higher abundance in lymph nodes compared to bone marrow and spleen. Interestingly, the frequency of F-MuLV-infected cells and the frequency of SFFV-infected cells were comparable, and single-infected cells clearly dominated over double-infected cells in this early phase. For most cell types, the frequencies of single-infected cells were ~ 10- to 100-fold higher than the frequency of double-infected cells, with the notable exception of myeloid cells, and Gr1^−^ myeloid cells in particular, which exhibited comparable frequencies of double-infected and single-infected cells already on day 4.

From day 7 and more pronounced on day 10, the frequency of double-infected cells increased to levels similar to those of single-infected cells. On day 7, this was most prominent in the spleen, but it was also observed in the bone marrow and lymph nodes at the later time points. The cell types showing the highest frequency of F-MuLV and SFFV infection in the late phase were erythroblasts, B cells and myeloid cells in the bone marrow, erythroblasts and B cells in the spleens, and erythroblasts, B cells and CD4^+^ T cells in the lymph nodes. It is striking to observe that from day 10, the infected cells in the spleen made up not only a small fraction, but in some cases even the majority of some of the analyzed cell types. This was not only true for erythroblasts, which massively expand in FV infection of susceptible mice due to the erythropoietin-independent erythroblast proliferation induced by SFFV [5, 29], but also and even more so for B cells and Gr1^+^ myeloid cells.

### Induction of IL-10 in F-MuLV and SFFV target cells

**Fig. 4:**
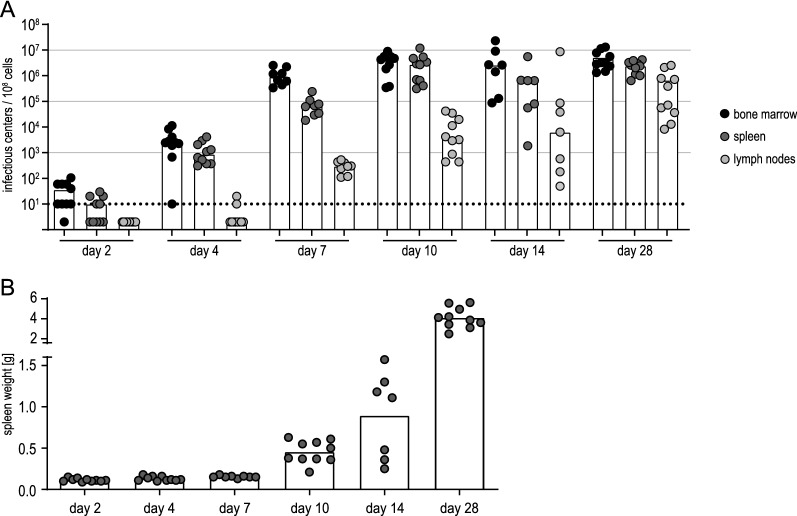
Infectious center frequencies in FV-Katushka-mTagBFP in BALB/c-based IL-10-eGFP reporter mice. Highly susceptible IL-10-eGFP mice of BALB/c background were infected with 500 SFFU of FV-Katushka-mTagBFP and viral loads in the bone marrow, lymph nodes and spleen on days 2, 4, 7, 10, 14 and 28 (**A**) and spleen weights (**B**) were analyzed. Each dot indicates an individual mouse, bars indicate the median (**A**) or mean values (**B**). The dotted line indicates the detection limit. Data from 7 (day 14), 8 (day 7) or 10 (day 2, 4, 10 28) mice per group were obtained in 1–2 independent experiments per time point

To analyze the induction of IL-10 by FV infection, which has been suggested to play an important role in the immune response against retroviruses [27, 43], we next infected BALB/c-based IL-10-eGFP reporter mice and analyzed the expression of the eGFP reporter in infected and uninfected cells at different time points. To obtain a deeper understanding of the early phase of infection, we included an analysis 2 days after infection. Importantly, the infection of the IL-10-eGFP mice resulted in comparable levels of infectious centers in the bone marrow, spleens and lymph nodes (Fig. 4) as in the wild-type (wt) BALB/c mice (Fig. 2).

**Fig. 5:**
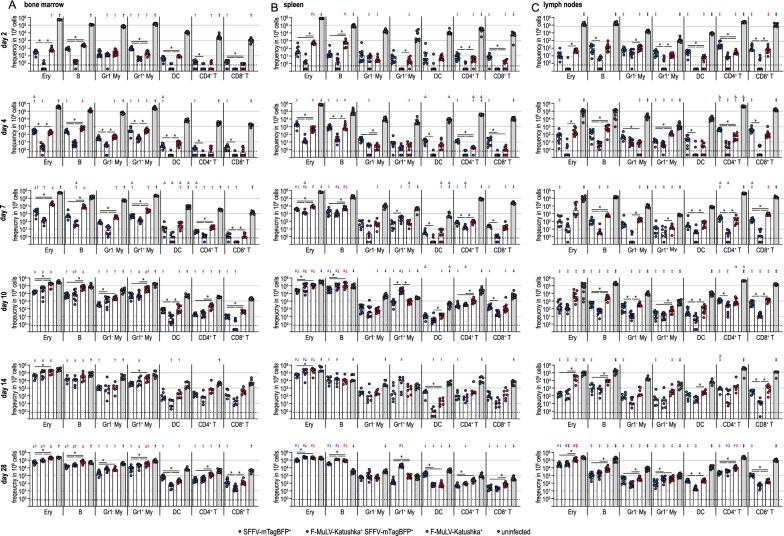
F-MuLV- and SFFV-infected cells in IL-10 reporter mice. Susceptible IL-10-eGFP reporter mice of BALB/c background were infected with 500 SFFU of FV-Katushka-mTagBFP and infected erythroblasts (Ery), B cells, Gr1^−^ (Gr1^−^ My) and Gr1^+^ myeloid cells (Gr1^+^ My), dendritic cells (DC), CD4^+^ T cells and CD8^+^ T cells in the bone marrow (**A**), spleen (**B**) and lymph nodes (**C**) were analyzed by flow cytometry on days 2, 4, 7, 10, 14 and 28. The frequencies of F-MuLV-Katushka single-infected, SFFV-mTagBFP single-infected and F-MuLV-Katushka, SFFV-mTagBFP double-infected as well as uninfected cells are shown as indicated. Each dot indicates an individual mouse, bars indicate median values, the dotted line indicates the detection limit. Data from 7 (day 14), 8 (day 7) or 10 (day 2, 4, 10, 28) mice per group were obtained in 1–2 independent experiments per time point. Asterisk indicates statistically significant differences between the indicated types of single or double-infected cells (*p* < 0.05, multiple t-test with Bonferroni correction for multiple comparisons). Number sign indicates dominant subsets of single or double-infected cells in the respective organ, i.e. infected cell types that were significantly higher than at least three other cell types of the same single or double-infected state (*p* < 0.05, Kruskal–Wallis ANOVA on Ranks, Dunn’s post test). Dagger indicates statistically significant differences between the respective cell subsets in the bone marrow and spleen, double dagger indicates statistically significant differences between the respective cell subsets in the bone marrow and lymph nodes, inverted cross symbol indicates statistically significant differences between the respective cell subsets in the spleen and lymph nodes (*p* < 0.05, multiple t-test with Bonferroni correction for multiple comparisons). Up-pointing triangle indicates statistically significant differences compared with the respective cell subsets in BALB/c mice (*p* < 0.05, multiple t-test with Bonferroni correction for multiple comparisons)

Similarly, when we analyzed the infected cell subsets in the bone marrow, spleens and lymph nodes of the infected IL-10-eGFP reporter mice, we found a highly comparable picture to that observed in wt BALB/c mice (Fig. 5). The additional analysis of cells expressing F-MuLV-Katushka or SFFV-mTagBFP proviruses on day 2 after infection revealed slightly lower levels of infected cells on day 2 compared to day 4, but already at this earliest time point we found low frequencies of double-infected cells, which in myeloid cells were at a level comparable to single-infected cells, as observed before in BALB/c mice on day 4.

**Fig. 6:**
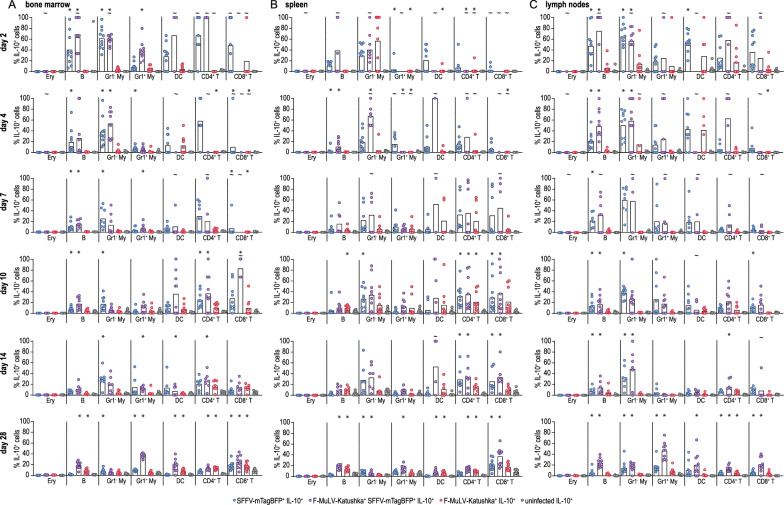
eGFP expression in infected and uninfected cells in IL-10-eGFP reporter mice. The infected and uninfected cells shown in Fig. 5 were analyzed for the expression of eGFP as an indicator of IL-10 expression. The frequency of eGFP^+^ cells in the respective cell population is shown. Each dot indicates an individual mouse, bars indicate median values. Tilde indicates infected cell subsets for which no percentage of eGFP^+^ cells could be calculated for one or more mice as no infected cells were detected. Data from 7 (day 14), 8 (day 7) or 10 (day 2, 4, 10 28) mice per group were obtained in 1–2 independent experiments per time point. Asterisk indicates statistically significant differences between the indicated infected types of single or double-infected cells compared to uninfected cells (*p* < 0.05, Kruskal–Wallis One Way ANOVA on Ranks, Dunn’s post test)

To investigate to what extent FV-infected cells contribute to the production of IL-10, we analyzed the frequency of eGFP^+^ cells in the subsets of single-infected, double-infected or uninfected cells (Fig. 6). In the early phase of infection, we observed IL-10 expression predominantly in B cells and myeloid cells in the bone marrow and spleen (Fig. 6A, B), whereas dendritic cells and T cells also contributed significantly to the total IL-10 expression in lymph nodes (Fig. 6C). The IL-10 expression pattern changed during the course of infection, and B cells, T cells, myeloid cells and dendritic cells contributed to the IL-10-expressing cells in all organs from day 7. Interestingly, the proportion of IL-10-expressing infected cells was significantly higher for many cell types than the proportion of IL-10-expressing uninfected cells, and infection with SFFV or double infection with both SFFV and F-MuLV induced a disproportionately high percentage of IL-10-expressing cells. While the frequency of IL-10-expressing cells in the uninfected cell populations was lower, it still must be considered that due to the higher overall cell numbers compared to the infected cell subsets, they contribute a substantial number of IL-10-expressing cells to the overall IL-10-expressing cell pool.

### Impact of IL-10 inactivation in T cells on FV infection levels and infected cell frequencies

**Fig. 7:**
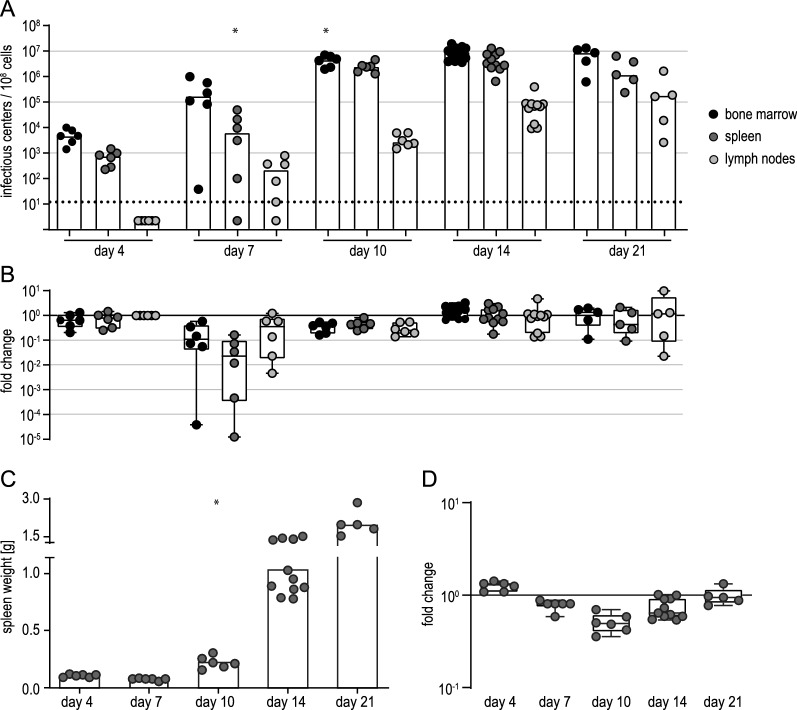
Influence of IL-10 inactivation in T cells on infectious center frequencies and splenomegaly. Tc-IL-10ko mice were infected with 500 SFFU of FV-Katushka-mTagBFP, and viral loads in the bone marrow, lymph nodes and spleen on days 4, 7, 10, 14 and 21 (**A**) and spleen weights (**C**) were analyzed. The fold-change compared to the mean viral loads (**B**) and mean spleen weights (**D**) of BALB/c mice as shown in Fig. 2 were calculated for visualization. Each dot indicates an individual mouse, bars indicate median (**A**) or mean values (**C**), the dotted line indicates the detection limit. Data from 5 (day 21), 6 (days 4, 7, 10) or 11 (day 14) mice per group were acquired in 1–3 independent experiments. Asterisk indicates statistically significant differences compared to the data of BALB/c mice as shown in Fig. 2 [data were collected side-by-side; *p* < 0.05, t test with Bonferroni correction for multiple comparisons (**A**, **C**)]

The immune control of FV in highly susceptible BALB/c mice is generally regarded as very weak. The finding that IL-10 is induced very early in FV infection suggested that in the absence of an IL-10-producing cell subset, immune control of FV infection might be improved, resulting in reduced levels of FV infection. We demonstrated in co-immunization experiments that the suppression of CD8^+^ T cell induction by F-MuLV Env was alleviated in mice with an ablation of IL-10 expression in T cells [44]. To verify the hypothesis that we would similarly observe improved control of FV infection in the absence of T cell produced IL-10, we infected BALB/c mice with an ablation of IL-10 expression in T cells (Tc-IL-10ko) with FV-Katushka-mTagBFP and compared the infection kinetics in these Tc-IL-10ko mice to the kinetics in BALB/c mice. We therefore analyzed the viral loads in the bone marrow, spleens and lymph nodes at different time points by infectious center assay and the infected target cells by flow cytometry as before, starting at day 4. When we analyzed the viral loads, we found that infectious center numbers were comparable between BALB/c and Tc-IL-10ko mice on day 4, but we did observe significant differences in viral loads on day 7 and day 10 when viral loads were lower in Tc-IL-10ko mice (Fig. 7A, B). At the later time points of day 14 and day 21 after infection, the viral loads were highly comparable, and no difference could be observed any longer. Similarly, the degree of splenomegaly was slightly reduced in Tc-IL-10ko mice compared to BALB/c mice between day 7 and day 14, with significantly lower spleen weights in Tc-IL-10ko mice on day 10 (Fig. 7C, D).

**Fig. 8:**
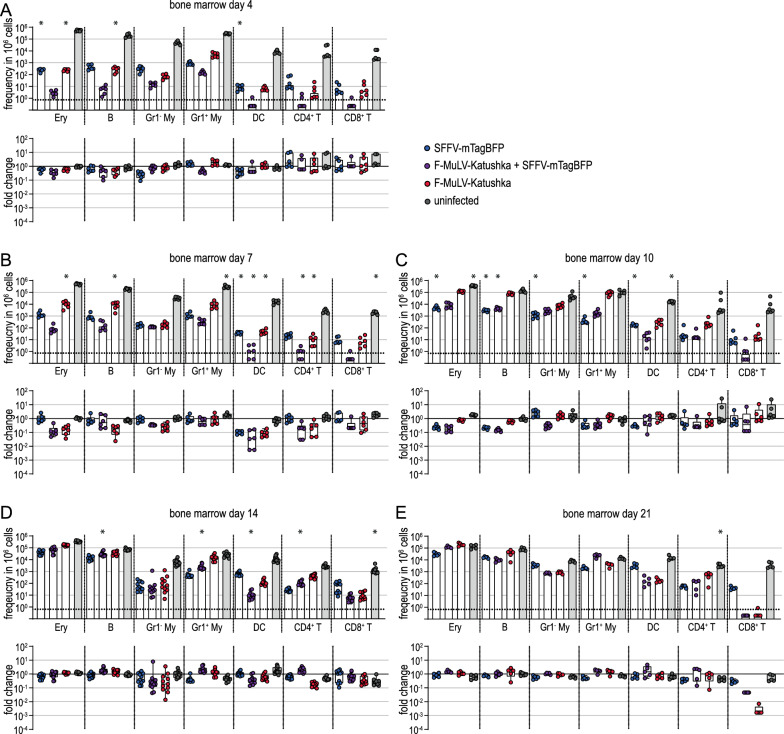
Influence of IL-10 inactivation in T cells on infected cells in the bone marrow. Tc-IL-10ko mice were infected with 500 SFFU of FV-Katushka-mTagBFP and infected erythroblasts (Ery), B cells, Gr1^−^ (Gr1^−^ My) and Gr1^+^ myeloid cells (Gr1^+^ My), dendritic cells (DC), CD4^+^ T cells and CD8^+^ T cells in the bone marrow were analyzed by flow cytometry on days 4 (**A**), 7 (**B**), 10 (**C**), 14 (**D**), and 21 (**E**). The frequencies of F-MuLV-Katushka single-infected, SFFV-mTagBFP single-infected and F-MuLV-Katushka, SFFV-mTagBFP double-infected as well as uninfected cells are shown in the upper graphs, and the fold-change calculated using the mean values obtained for BALB/c mice as shown in Fig. 3 is shown in the lower graphs. Data from 5 (day 21), 6 (days 4, 7, 10) or 11 (day 14) mice per group were acquired in 1 to 3 independent experiments. Asterisk indicates statistically significant differences compared to the data of BALB/c mice as shown in Fig. 3 [data were collected side-by-side; *p* < 0.05, t test with Bonferroni correction for multiple comparisons (upper graphs)]

**Fig. 9:**
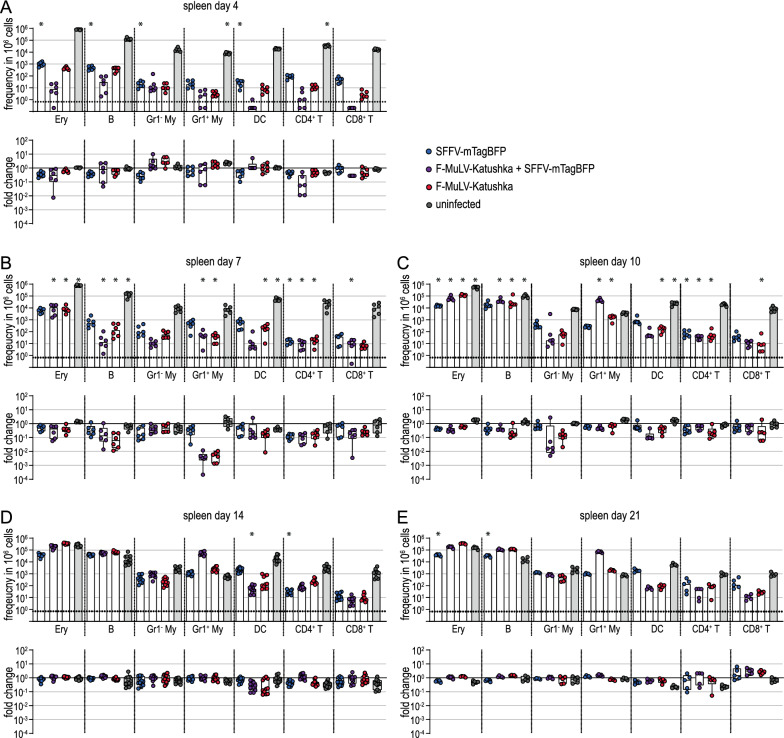
Influence of IL-10 inactivation in T cells on infected cells in the spleen. Tc-IL-10ko mice were infected with 500 SFFU of FV-Katushka-mTagBFP and infected erythroblasts (Ery), B cells, Gr1^−^ (Gr1^−^ My) and Gr1^+^ myeloid cells (Gr1^+^ My), dendritic cells (DC), CD4^+^ T cells and CD8^+^ T cells in spleens were analyzed by flow cytometry on days 4 (**A**), 7 (**B**), 10 (**C**), 14 (**D**), and 21 (**E**). The frequencies of F-MuLV-Katushka single-infected, SFFV-mTagBFP single-infected and F-MuLV-Katushka, SFFV-mTagBFP double-infected as well as uninfected cells are shown in the upper graphs, and the fold-change calculated using the mean values obtained for BALB/c mice as shown in Fig. 3 is shown in the lower graphs. Data from 5 (day 21), 6 (days 4, 7, 10) or 11 (day 14) mice per group were acquired in 1 to 3 independent experiments. Asterisk indicates statistically significant differences compared to the data of BALB/c mice as shown in Fig. 3 [data were collected side-by-side; *p* < 0.05, t test with Bonferroni correction for multiple comparisons (upper graphs)]

**Fig. 10:**
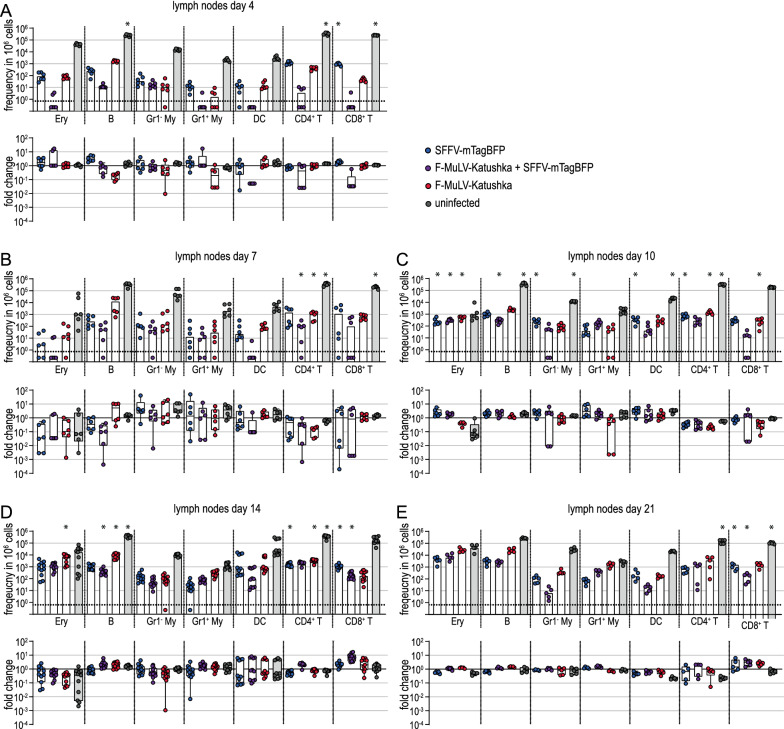
Influence of IL-10 inactivation in T cells on infected cells in lymph nodes. Tc-IL-10ko mice were infected with 500 SFFU of FV-Katushka-mTagBFP and infected erythroblasts (Ery), B cells, Gr1^−^ (Gr1^−^ My) and Gr1^+^ myeloid cells (Gr1^+^ My), dendritic cells (DC), CD4^+^ T cells and CD8^+^ T cells in lymph nodes were analyzed by flow cytometry on days 4 (**A**), 7 (**B**), 10 (**C**), 14 (**D**), and 21 (**E**). The frequencies of F-MuLV-Katushka single-infected, SFFV-mTagBFP single-infected and F-MuLV-Katushka, SFFV-mTagBFP double-infected as well as uninfected cells are shown in the upper graphs, and the fold-change calculated using the mean values obtained for BALB/c mice as shown in Fig. 3 is shown in the lower graphs. Data from 5 (day 21), 6 (days 4, 7, 10) or 11 (day 14) mice per group were acquired in 1 to 3 independent experiments. Asterisk indicates statistically significant differences compared to the data of BALB/c mice as shown in Fig. 3 [data were collected side-by-side; *p* < 0.05, t test with Bonferroni correction for multiple comparisons (upper graphs)]

When we analyzed the infected cell subsets in detail by flow cytometry, we found some differences in infected cell numbers on day 4 after infection in the bone marrow (Fig. 8A), spleen (Fig. 9A) and lymph nodes (Fig. 10A). In spleens, the frequency of SFFV-infected erythroblasts, B cells, Gr1^−^ myeloid cells and DCs was significantly lower in Tc-IL-10ko mice than in BALB/c mice. In the bone marrow, the effect was slightly less pronounced, but the frequency of SFFV or F-MuLV single-infected erythroblasts, of F-MuLV-infected B cells and SFFV-infected DCs was similarly lower in Tc-IL-10ko mice. On the other hand, infected target cell frequencies were highly comparable in the lymph nodes, with the exception of SFFV-infected CD8^+^ T cells, which were slightly but significantly higher in Tc-IL-10ko mice.

On day 7 and day 10 after infection, the differences were again most pronounced in the spleen cells (Fig. 9B, C), and the most pronounced difference was observed for Gr1^+^ myeloid cells on day 7, when the levels of F-MuLV-infected or double-infected Gr1^+^ myeloid cells were lower in Tc-IL-10ko mice by approximately 2 log. We found significantly lower levels of SFFV or F-MuLV single- and double-infected erythroblasts, where the frequency was approximately twofold higher in BALB/c mice than in Tc-IL-10ko mice. Furthermore, the frequency of single or double-infected cells was also lower in B cells, Gr1^−^ myeloid cells, DCs and CD4^+^ and CD8^+^ T cells. The differences were less pronounced in bone marrow (Fig. 8B, C), where we found the most pronounced differences in the frequency of infected erythroblasts and B cells. Interestingly, we observed slightly lower frequencies of infected cells in CD4^+^ T cells in the lymph nodes of Tc-IL-10ko mice compared to BALB/c mice on day 7 and day 10 (Fig. 10B, C). In contrast, the frequency of SFFV-, F-MuLV- or double-infected erythroblasts, SFFV or double-infected B cells, and SFFV-infected Gr1^−^ myeloid cells and DCs was slightly but significantly higher in Tc-IL-10ko mice than in BALB/c mice in lymph nodes on day 10 (Fig. 10C).

In the late phase of infection, the differences in infected target cell frequencies between Tc-IL-10ko mice and BALB/c mice were less pronounced (Figs. 8D, E, 9D, E and 10D, E), although there were still slightly lower frequencies in SFFV-infected erythroblasts, B cells and DCs in spleens on day 21 (Fig. 10E).

### Increased T cell activation in Tc-IL-10ko mice

**Fig. 11:**
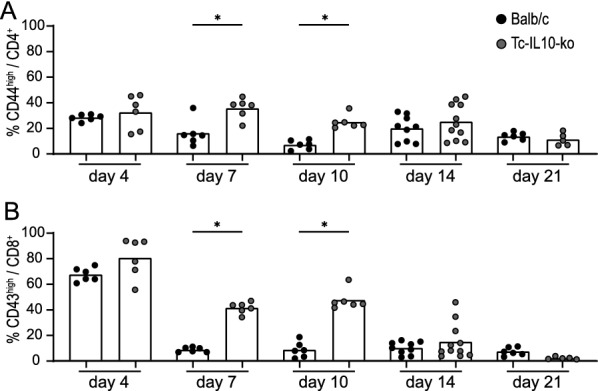
Activation of CD4^+^ and CD8^+^ T cells in Tc-IL-10ko mice. BALB/c or Tc-IL-10ko mice were infected with 500 SFFU of FV-Katushka-mTagBFP and activated CD4^+^ T cells (**A**) and CD8^+^ T cells (**B**) in spleens were analyzed by flow cytometry on days 4, 7, 10, 14, and 21. Each dot indicates an individual mouse, bars indicate the mean values. Data from 5 [day 21 (Tc-IL-10ko)], 6 [days 4, 7, 10, 21 (BALB/c)], 9 [day 14 (BALB/c)] or 11 [day 14 (Tc-IL-10ko)] mice per group were acquired in 1 to 3 independent experiments. Asterisk indicates statistically significant differences between the frequencies of the respective cell subsets in BALB/c and Tc-IL-10ko mice (*p* < 0.05, Student’s t test)

Little is known about the T cell responses in BALB/c mice, and no CD8^+^ T cell epitopes of FV have been identified for this mouse strain, but the presence of H-2D^d^- or H-2L^d^-restricted cytotoxic T cell responses against FV has been indicated by cytotoxicity assays using H-2^d^ leukemia target cells [45]. To analyze the impact of IL-10 ablation in T cells on T cell responses, we therefore analyzed the activation patterns of CD4^+^ and CD8^+^ T cells in FV-Katushka-mTagBFP infected Tc-IL-10ko mice in comparison to BALB/c mice (Fig. 11). At the early time point of day 4 after FV-Katushka-mTagBFP infection, we observed only a trend toward a higher activation rate of CD4^+^ T cells (Fig. 11A) and CD8^+^ T cells (Fig. 11B). These differences were very pronounced on day 7 and day 10 after infection when differences in infected cell frequencies were also most pronounced, with significantly higher frequencies of activated CD4^+^ and CD8^+^ T cells in Tc-IL-10ko mice compared to BALB/c mice. In the late phase of the infection on day 14 and day 21, no differences in CD4^+^ and CD8^+^ T cell activation could be observed any longer, which correlates with the fact that we only observed minor differences in the frequency of infected cells at these time points.

## Discussion

In this study, we present a new double fluorescently labeled FV complex that allows for the individual analysis of F-MuLV and SFFV-infected cells by flow cytometry. Importantly, we did not observe any overall changes in the pathogenicity of our double fluorescently labeled FV-Katushka-mTagBFP, since mice developed splenomegaly and had sustained high viral loads from day 10, which is the expected outcome of FV infection of highly susceptible mice [42]. However, disease development was delayed compared to unmodified, uncloned FV, which may be due to the fluorescent transgenes or to differences between the particular SFFV and F-MuLV clones used in the modified complex and those sequences in the uncloned FV. The fact that disease development was delayed rather than abrogated and that mice developed severe splenomegaly and high infection levels at the later time points hints at slightly impaired efficiency of SFFV replication or of SFFV gp55 interaction with EpoR or sf-Stk. However, the overall pathogenic effect of FV infection in highly susceptible mice has not been changed, and therefore, we are confident that our findings still provide an accurate picture of the infected target cell populations. We previously used an FV complex containing mWasabi-labeled F-MuLV to characterize FV target cells, and showed that in addition to erythroblasts, myeloid cells and B cells and, to a lesser extent, T cells were important target cells for FV infection of resistant and susceptible mice [34]. The findings with our new double labeled FV-Katushka-mTagBFP confirm these results and show that the distribution of F-MuLV and SFFV is very similar, which can be readily explained by the fact that SFFV relies on the proteins of its helper virus F-MuLV for dissemination [11]. Other studies performed previously with non-labeled FV also demonstrated predominant infection of erythroblasts, B cells and myeloid cells [46–49]. We therefore conclude that the results obtained with our fluorescently labeled viruses are very much in line with previous knowledge and that the fluorescent label, which by fusion via a 2 A self-cleaving peptide is produced as a separate protein [50] that is not directed toward incorporation into the virus particles and labels the infected cell rather than the virion, does not affect target cell tropism.

As we have discussed previously [34], the preference of FV for the infection of erythroblasts, myeloid cells and B and T cells can be explained on the one hand by high expression levels of the cellular FV receptor mCat-1 [51], the high basal proliferation rate in the case of erythroblasts, and the nature of myeloid cells as important antigen-presenting cells. As such, myeloid cells have a decidedly scavenging phenotype and exhibit increased permissiveness for infection by many pathogens, which allows for increased viral loads in these cells and thus an improved induction and orchestration of immune responses [52].

It should be noted that the fluorescent intensity of the infected cells was variable; however, we did not make a distinction of more or less brightly fluorescent cells. While a stronger fluorescent intensity might be indicative of the expression of multiple proviruses, other factors, such as the availability of host transcription factors and the chromatin environment, can also influence individual provirus expression levels (reviewed in [53]).

In the infection of highly susceptible mice, such as the BALB/c and BALB/c-based mice used in the experiments presented here, the proliferation rate of erythroblasts is markedly increased due to the pathogenic effect of SFFV. In SFFV-infected erythroblasts of BALB/c mice, the SFFV envelope protein gp55 interacts with the erythropoietin receptor [54–57] and the truncated receptor tyrosine kinase sf-Stk [58–61], which results in the strongly increased, erythropoietin-independent proliferation of erythroblasts. This highly proliferating erythroblast population is a prime target for subsequent infection with F-MuLV, SFFV, or both, leading to an extremely high infection rate in the late phase of FV infection, when the majority of erythroblasts in spleens are infected by one or both viruses. Of note, infection with SFFV alone, which was observed far more frequently than double infection in the early phase of FV infection, is sufficient for the induction of erythroblast proliferation, since SFFV gp55 is also expressed in the absence of F-MuLV and will by itself interact with and activate the erythropoietin receptor and sf-Stk to induce erythroleukemia [29].

The differences in the frequency of single- and double-infected cells, which is most pronounced in the early phase of the infection, i.e. when the frequencies of infected cells are still relatively low, indicate that double-infected cells are indeed a result of two infection events, rather than the co-packaging of F-MuLV and SFFV genomes into the same virion. While the presence of F-MuLV in the same cell as SFFV is necessary for the production of SFFV virions, its presence is not required in the target cell for reverse transcription, integration and expression of the SFFV genome. Cells infected with SFFV alone, however, only express SFFV genes without the production of infectious progeny SFFV particles.

It seems to be mainly the high viral loads of the later infection phase that drives the double infection of cells with both F-MuLV and SFFV, as these double-infected cells become more dominant as from day 10 in most cell types. One notable exception is the double infection of myeloid cells, which already occurs in the very early phase of the infection and can likely be attributed to the scavenging nature of this cell type. The requirement of two infection events for a double infection with F-MuLV and SFFV is in agreement with early findings when experiments of Fv-1-matched and unmatched mice showed that the induction of SFFV-induced spleen foci followed a one-hit kinetic in Fv-1 matched, and a two-hit kinetic in Fv-1 unmatched mice [62, 63]. While the sequences at the 5′ end of the genomes of F-MuLV and SFFV are sufficiently similar to possibly allow dimerization in that region, the genomes have distinctly different sizes due to the numerous deletions in the SFFV genome [11], which may limit the tendency of the two genomes to form hetero-dimers. Furthermore, the reverse transcriptase performs one or more template switches during the reverse transcription of the RNA genome into the DNA pregenome [64–67], implying that two different RNA genomes in one particle usually do not result in two DNA pregenomes in the infected cell. While our in vivo data strongly suggest that two infection events lead to double F-MuLV- and SFFV-infected cells, our data are not sufficient to rule out, or judge the rate of, the co-packaging of F-MuLV and SFFV. Intriguingly, we showed in in vitro infection experiments with a double fluorescently labeled FV complex that foci containing double-infected cells could also be observed when only a low virus inoculum was used and the resulting foci were spaced relatively far apart (Additional file 1: Fig. S2). The low infection dose reduced the likelihood of a double infection, so the double-positive foci obtained with a low virus inoculum could indeed hint at a certain low rate of co-packaging of F-MuLV and SFFV genomes. Further experiments will be performed in the future to address this question in more detail.

The induction of IL-10 that we observed in the FV-Katushka-mTagBFP infected mice could be detected already in the early phase of infection and was as pronounced in SFFV single or F-MuLV/SFFV double-infected cells as in F-MuLV-infected cells. Interestingly, we observed differences in the predominant IL-10-expressing cell types between the different organs, and changes within organs over time. This is likely due on the one hand to different cell subtypes, e.g. different myeloid cell populations, being present in different organs such as in the spleen compared to in the bone marrow (reviewed in [68]), whereas changes over time are likely due to changes in the cytokine milieu induced by the ensuing immune response.

The contribution of SFFV-infected cells to the IL-10-producing cell pool is a surprising finding since SFFV has not been implied in the immunosuppression by IL-10 induction before. While the induction of IL-10 has been described before for various retrovirus infections, it has been attributed to the immunosuppressive domain (ISD), which is located in the retroviral transmembrane envelope protein [43, 69, 70]. In the past, we observed in immunization experiments that F-MuLV Env has the capacity to suppress CD8^+^ T cell responses to simultaneously applied immunogens [19, 27]. In SFFV, however, the Env ISD is not present due to a large deletion in the SFFV Env sequence [71]. We confirmed by in vitro stimulation experiments that both F-MuLV and SFFV induce the production of IL-10 by spleen cells, corroborating the flow cytometry data (Additional file 1: Fig. S3). Since the ISD is not present in SFFV gp55, it is intriguing to speculate that the interaction of SFFV gp55 with sf-Stk, which has mainly been described for its important role in promoting erythroblast proliferation by SFFV [4, 58], might contribute to the induction of IL-10. Stk is encoded by the *ron* gene and is the murine homolog of the human Ron kinase [72]. A synonym of Stk is macrophage stimulating 1 receptor (Mst1R); it is the receptor for macrophage stimulating protein (MSP) and belongs to the family of c-Met-related tyrosine kinases [73]. For human Ron as well as murine Stk, a role in the activation of cytokine and effector molecule expression by macrophages has been demonstrated [74–77], implying that Stk/Ron is an important regulator of immune responses. Whether the interaction of sf-Stk with SFFV gp55 similarly influences immune responses, which might then result in increased IL-10 production, has not been investigated until now. It is important to note that Stk/sf-Stk expression is not limited to erythroblasts and macrophages but has been demonstrated for various developmental stages of hematopoietic cells [72] and endothelial cells [78]. Whether B cells and T cells also express Stk/sf-Stk has not been conclusively demonstrated. The interaction of SFFV gp55 with sf-Stk induces sf-Stk activation and intracellular signaling that results in cell proliferation [58], and the co-expression of gp55 and sf-Stk has been shown to be sufficient for the induction of cell proliferation [60].

A specific interference of SFFV with immunity in BALB/c mice and an involvement of sf-Stk is plausible, since this mouse strain mounts notoriously weak immune responses against FV [42]. We demonstrated in our experiments that inactivation of IL-10 in T cells resulted in reduced virus levels in the intermediate phase of infection. While no specific CD4^+^ or CD8^+^ T cell epitopes have been described for FV in H-2^d^ mice, we observed stronger CD4^+^ and CD8^+^ T cell activation in Tc-IL-10ko mice in the intermediate phase of infection, indicating that the BALB/c mice mount a cellular immune response with the potential of controlling FV infection to a certain, although limited degree. We already observed reduced frequencies of infected cells in Tc-IL-10ko mice at the early time point of 4 days after infection. This suggests that besides the CD4^+^ and CD8^+^ T cell response, the response of innate cells might also be improved in IL-10ko mice, such as the response by natural killer cells, which have been shown to be involved in the control of FV infection [79, 80]. The strong stimulation of erythroblast proliferation and the rise of fully transformed erythroleukemia cells lead to very high levels of FV replication and limit the extent of immune control. Furthermore, since we abrogated IL-10 expression only in T cells, other cell types still expressed IL-10, and a more complete block of IL-10 might result in more pronounced effects.

## Conclusion

Overall, our results provide valuable novel insight into FV infection in susceptible mice and suggest the influence of SFFV gp55 on immune responses and on infected target cells other than erythroblasts as an interesting focus for future research. It will be of great interest to investigate the role of SFFV in FV infection in more detail in the future.

## Methods

### Cells

A murine fibroblast cell line from *Mus dunni* [81] and the murine hybridoma cell line 720 [82] were maintained in RPMI medium supplemented with 10% heat-inactivated fetal bovine serum, 50 µg/ml gentamicin and 20 µg/ml ciprofloxacin. The human embryonic kidney cell line 293T was maintained in DMEM supplemented with 10% heat-inactivated fetal bovine serum, 50 µg/ml gentamicin and 20 µg/ml ciprofloxacin (all cell culture reagents were obtained from Invitrogen/Gibco, ThermoFisher Scientific, Karlsruhe, Germany). Cells were maintained in a humidified 5% CO_2_ atmosphere at 37 °C.

### Unmodified Friend virus

Uncloned, lactate dehydrogenase-elevating virus (LDV)-free FV stock was obtained from BALB/c mouse spleen cell homogenate (10%, wt/vol) 14 days post infection with a B-tropic, polycythemia-inducing FV complex as described previously [83].

### Fluorescently labeled virus cloning and reconstitution

A modified F-MuLV was created that encodes the red fluorescent protein Katushka [38] fused to the C terminus of the envelope open reading frame linked by the self-cleaving 2A peptide of porcine teschovirus [50] (Fig. 1A). Cloning was performed using plasmid pFB29, which encodes a permuted clone of F-MuLV strain FB29 [84] (pFB29 kindly provided by Marc Sitbon, Institut Génétique Moléculaire de Montpellier, Montpellier, France; kindly transferred by Masaaki Miyazawa, Kindai University Faculty of Medicine, Osaka, Japan). The coding sequence for Katushka was amplified by PCR, and flanking restriction sites for BglII and XbaI were introduced for cloning into pBlu.p15E-2A-Wasabi-U3, replacing the mWasabi coding sequence in the previously described plasmid [34]. The ClaI-AscI fragment of this plasmid containing the C-terminus of p15E, 2A peptide, Katushka, and U3 was introduced into pFB29 with ClaI and AscI. For reconstitution of the Katushka-encoding F-MuLV-Katushka, the genome was released from the pFB29-2A-Katushka plasmid by HindIII digestion, religated, and transfected into 293T cells. The recovered virus was purified from the supernatants of transfected 293T cells and passaged on Mus dunni cells, and the virus was purified by sucrose cushion ultracentrifugation.

A modified SFFV was created that encodes the blue fluorescent protein mTagBFP [39] fused to the C-terminus of the envelope open reading frame linked by a self-cleaving 2A peptide, analogous to the design of the modified F-MuLV. In preparation for cloning, NheI and SalI restriction sites were introduced by site-directed mutagenesis into the previously described plasmid pMK-SFFV, which contains a colinear molecular clone of polycythemic SFFV [34], replacing the Env stop codon. The mTagBFP coding sequence was amplified by PCR, and flanking restriction sites for BglII and XbaI were introduced for cloning into pBlu.p15E-2A-Wasabi-U3, replacing the mWasabi coding sequence in the previously described plasmid [34]. The 2A-mTagBFP sequences were again amplified by PCR, and flanking NheI and SalI restriction sites were introduced. 2A-mTagBFP was subsequently cloned into the modified pMK-SFFV using the NheI and SalI restriction sites.

To reconstitute the complex of F-MuLV-Katushka and SFFV-mTagBFP, we transfected pMK-SFFV-2A-mTagBFP into *Mus dunni* cells and superinfected the cells with F-MuLV-Katushka. The complex was passaged on *Mus dunni* cells, and virus stocks were prepared as described above. To obtain an in vivo stock of the FV-Katushka-mTagBFP complex, BALB/c mice were infected with the purified complex; spleens were collected 15 days after infection when splenomegaly was established, and 15% spleen cell homogenates were prepared and stored at **− **80 °C. The infectious titer of the stock, expressed as spleen focus-forming units (SFFU) per milliliter of virus stock, was determined by infection of CB6F1 mice and the determination of spleen foci 14 days after infection using fixation by Bouin’s fixative as described previously [85].

### Mice and FV infection

BALB/c mice were purchased from Charles River (Erkrath, Germany). BALB/c background IL-10-eGFP reporter mice carry an IRES-eGFP cassette in the intron behind exon 5 of the IL-10 coding sequence ([86]; mice had been backcrossed to BALB/c at least 12 times). Mice with a conditional knock-out of IL-10 in T cells (Tc-IL-10ko) were obtained by crossing the BALB/c background strains IL-10tm1Roer (carrying loxP sites upstream of the IL-10 promoter and downstream of the IL-10 exon1 [87]; mice had been backcrossed to BALB/c at least 12 times; kindly provided by Axel Roers, Institute of Immunology, Technical University Dresden, now Institute of Immunology, University Hospital Heidelberg, Germany) and Tg(CD4-cre) (expressing Cre recombinase under control of CD4 enhancer, promoter and silencer sequences [88]; mice had been backcrossed to BALB/c at least 12 times). All mice were at least 8 weeks old at the onset of the experiments. Both male and female mice were used in the experiments.

Mice were infected with 500 SFFU of FV-Katushka-mTagBFP in 100 µl of PBS by intravenous injection into the tail vein. For the analysis of infected cells, mice were sacrificed at different time points after infection, and the spleens, lymph nodes (axillary, brachial, cervical, inguinal and popliteal), and bone marrow (femoral and tibial) were collected.

All mouse experiments were performed with permission from the authorities (North Rhine-Westphalia State Office for Nature, Environment and Consumer Protection, LANUV NRW) and in accordance with the national law and the institutional guidelines of the University Hospital Essen, Essen, Germany.

### Analysis of infectious centers

Dilutions of single-cell suspensions of spleen, lymph node, and bone marrow cells were seeded onto *Mus dunni* fibroblasts and incubated for 3 days until cells reached confluence. Infected cells were detected by immunocytochemical staining using the hybridoma-derived antibody 720 as described previously [41].

### Flow cytometric analysis of infected cells

Single-cell suspensions of spleen, lymph node, and bone marrow cells were stained with the following antibody panel: PerCP5.5-anti-CD21/CD35, APC-anti-CD11c, AF700-anti-CD19, BV510-anti-CD45R/B220, BV605-anti-CD11b, BV650-anti-IgD, BV711-anti-CD44, BV785-anti-Ter119, PE/Cyanine5-anti-Ly-6G/Ly-6C (GR-1), PE/Cyanine7-anti-CD23 (BioLegend, San Diego, California, United States), BV750-anti-CD5, BUV395-anti-CD4, BUV496-anti-CD3e, BUV563-anti-CD43, BUV615-anti-CD8a, BUV661-anti-CD93, and BUV737-anti-IgM (Becton-Dickinson, Franklin Lakes, United States).

Data were acquired on a BD FACSymphony A5 flow cytometer (Becton-Dickinson) and analyzed using FlowJo software (TreeStar, Ashland, Oregon, United States).

### Statistical analysis

Analysis of data for statistically significant differences was performed either using an unpaired t test for the comparison of parametric data from two groups or using a nonparametric one-way analysis of variance on ranks with Dunn’s multiple-comparison procedure in GraphPad Prism 8 software for the comparison of more than two groups.

## Supplementary Information

**Additional file 1: Figure S1.** Comparison of uncloned FV complex and FV-Katushka-mTagBFP. **Figure S2.** Analysis of single- and double-infected foci by fluorescence microscopy. **Figure S3.** Induction of IL-10 by in vitro stimulation.

## Data Availability

The datasets used and/or analyzed during the current study are available from the corresponding author on reasonable request.
