# Supplementary material for: A detailed analysis of F-MuLV- and SFFV-infected cells in Friend virus-infected mice reveals the contribution of both F-MuLV- and SFFV-infected cells to the interleukin-10 host response
Source: Retrovirology. 2022 Dec 16;19:29. doi: 10.1186/s12977-022-00613-4 (PMC9758943; doi:10.1186/s12977-022-00613-4)
Supplement: Supplementary file 1 — Additional file 1: Figure S1. Comparison of uncloned FV complex and FV-Katushka-mTagBFP. Figure S2. Analysis of single- and double-infected foci by fluorescence microscopy. Figure S3. Induction of IL-10 by in vitro stimulation. [file 12977_2022_613_MOESM1_ESM.pdf]

Figure S1

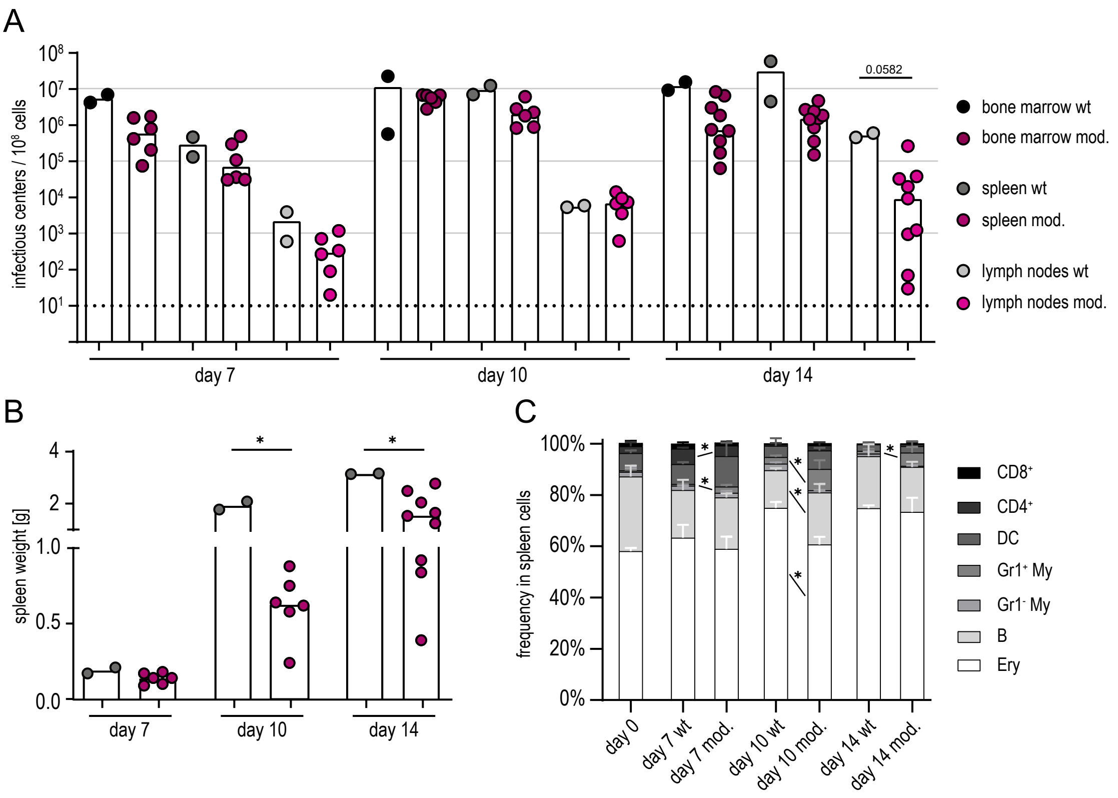

Comparison of uncloned FV complex and FV-Katushka-mTagBFP

For a comparison of the infection kinetics, BALB/c mice were infected with uncloned B-tropic FV complex and the number of infectious centers in bone marrow, spleen and lymph nodes (A), spleen weights (B) and cell type frequencies in spleens (C) were analyzed at the indicated time points. The data for the modified FV-Katushka-mTagBFP (mod.) are the same as in Figure 2 and are shown for reference. No statistically significant differences were observed for the frequencies of infectious centers (A; $p > 0.05$, Welch‘s t test). Significant differences in spleen weights were detected on day 14 (B; * $p < 0.05$, Welch‘s t test). The frequencies of the indicated cell subsets in splenocytes revealed statistically signifiant differences in frequencies of cell subsets with the largest differences on day 10 (C; $p < 0.05$, Welch‘s t test with Bonferroni correction for multiple comparisons). The frequency of erythroblasts in spleen cells was similar on day 14, indicating a comparable but delayed pathogenic effect on erythroblasts of both uncloned FV-B and FV-Katushka-mTagBFP.

Figure S2

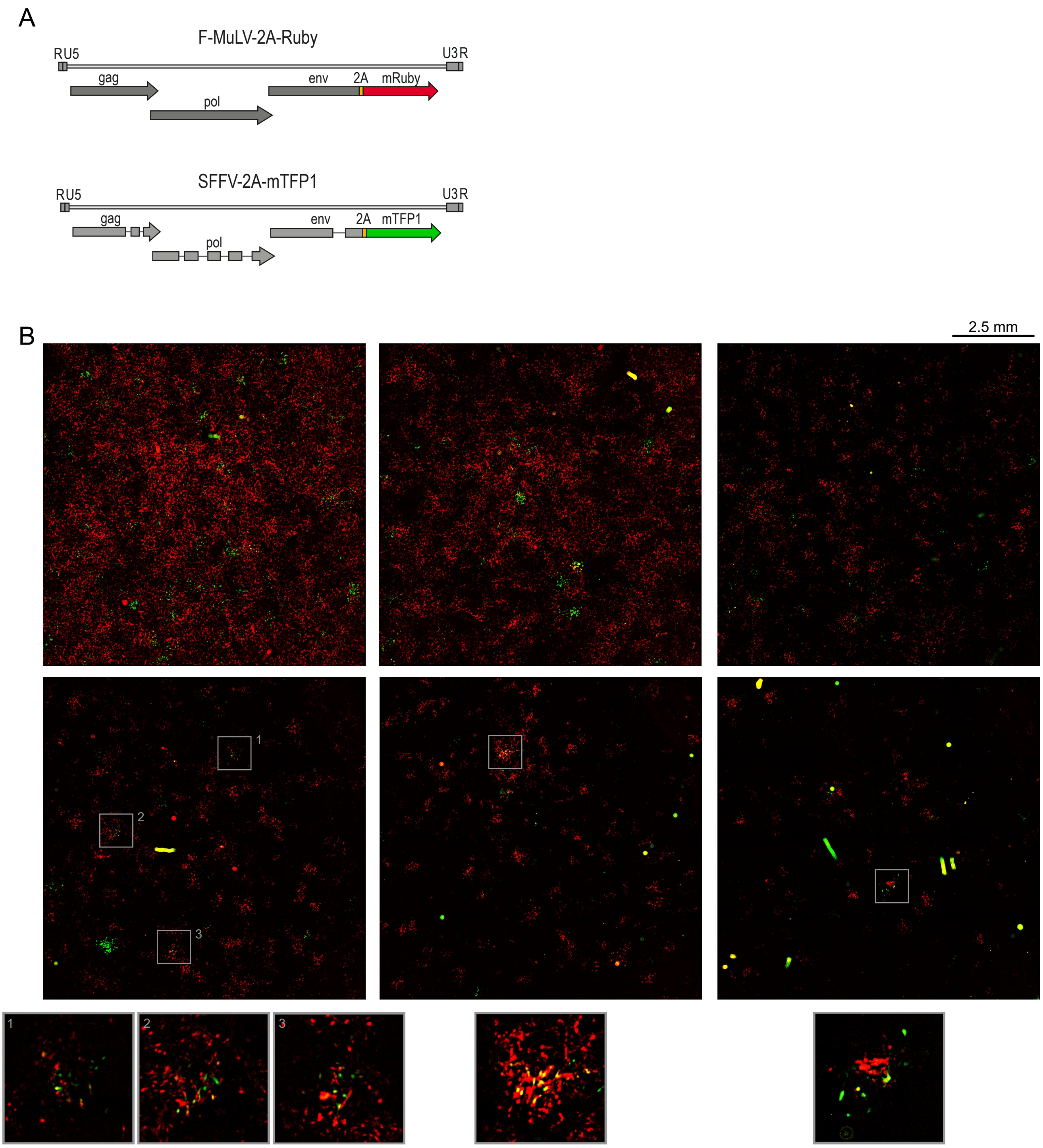

Analysis of single- and double-infected foci by fluorescence microscopy

For an analysis of single- and double-infected cells in infected *Mus dunni* fibroblast cells, cells were seeded in a 24-well plate and infected with serial dilutions of an FV complex consisting of F-MuLV-mRuby and SFFV-mTFP1 (A). Cells were imaged using a Leica Thunder microscope using 575 nm for excitation of mRuby and 440 nm for excitation of mTFP1 fluorescence. Images were obtained by merging multiple images obtained at 5x magnification, the scale bar indicates 2.5 mm (B). The gray boxes in the lower row pictures indicate foci that contain both green and red cells, which are shown at 20x magnification in the pictures below.

Figure S3

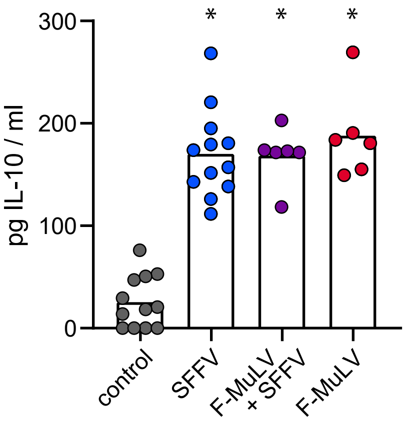

Induction of IL-10 by *in vitro* stimulation

For the *in vitro* analysis of IL-10 induction, 10^6 spleen cells from a BALB/c mouse were seeded onto 293T human embryonic kidney cells in 24-well plates that had been transfected 16 hours before with 1 µg of the SFFV-mTagBFP-containing plasmid pMK-SFFV-2A-mTagBFP together with 1 µg each of the F-MuLV Env encoding plasmid pCG env and the F-MuLV Gag-Pol encoding plasmid pcDNA.GagPol (SFFV), or with 1 µg of the HindIII digested and religated F-MuLV-Katushka from pFB-2A-Katushka (F-MuLV), or the combination of the two (F-MuLV+SFFV), or with expression plasmids of the fluorescent proteins Katushka or mTagBFP as control. The spleen cells were incubated on the transfected 293T cells for 48 hours and were then transferred to separate wells and incubated in 200 µl of fresh FCS-supplemented RPMI medium for another three days. Supernatants were collected and subjected to an IL-10 ELISA (ELISA MAX Standard Set Mouse IL-10, Biolegend, San Diego, CA). Significantly elevated levels of IL-10 were detected in the supernatants of spleen cells stimulated with SFFV, F-MuLV or F-MuLV and SFFV ($p < 0.05$ compared to control plasmid, One Way ANOVA with Tukey‘s multiple comparison test).
